# Extracellular DNA release, quorum sensing, and PrrF1/F2 small RNAs are key players in *Pseudomonas aeruginosa* tobramycin-enhanced biofilm formation

**DOI:** 10.1038/s41522-019-0088-3

**Published:** 2019-05-23

**Authors:** Ali Tahrioui, Rachel Duchesne, Emeline Bouffartigues, Sophie Rodrigues, Olivier Maillot, Damien Tortuel, Julie Hardouin, Laure Taupin, Marie-Christine Groleau, Alain Dufour, Eric Déziel, Gerald Brenner-Weiss, Marc Feuilloley, Nicole Orange, Olivier Lesouhaitier, Pierre Cornelis, Sylvie Chevalier

**Affiliations:** 1 Laboratoire de Microbiologie Signaux et Microenvironnement, LMSM EA4312, Normandie Université, Université de Rouen Normandie, Évreux, France; 2 Laboratoire Polymères Biopolymères Surfaces, PBS, Normandie Université, Université de Rouen Normandie, UMR 6270 CNRS, Mont Saint Aignan, France; 3 Laboratoire de Biotechnologie et Chimie Marines, LBCM EA3884, Institut Universitaire Européen de la Mer, Université de Bretagne-Sud, Lorient, France; 4 INRS-Institut Armand-Frappier, Laval, QC Canada; 5 Institute of Functional Interfaces, Karlsruhe Institute of Technology, Karlsruhe, Germany

**Keywords:** Biofilms, Antimicrobials, Pathogens

## Abstract

Biofilms are structured microbial communities that are the leading cause of numerous chronic infections which are difficult to eradicate. Within the lungs of individuals with cystic fibrosis (CF), *Pseudomonas aeruginosa* causes persistent biofilm infection that is commonly treated with aminoglycoside antibiotics such as tobramycin. However, sublethal concentrations of this aminoglycoside were previously shown to increase biofilm formation by *P. aeruginosa*, but the underlying adaptive mechanisms still remain elusive. Herein, we combined confocal laser scanning microscope analyses, proteomics profiling, gene expression assays and phenotypic studies to unravel *P. aeruginosa* potential adaptive mechanisms in response to tobramycin exposure during biofilm growth. Under this condition, we show that the modified biofilm architecture is related at least in part to increased extracellular DNA (eDNA) release, most likely as a result of biofilm cell death. Furthermore, the activity of quorum sensing (QS) systems was increased, leading to higher production of QS signaling molecules. We also demonstrate upon tobramycin exposure an increase in expression of the PrrF small regulatory RNAs, as well as expression of iron uptake systems. Remarkably, biofilm biovolumes and eDNA relative abundances in *pqs* and *prrF* mutant strains decrease in the presence of tobramycin. Overall, our findings offer experimental evidences for a potential adaptive mechanism linking PrrF sRNAs, QS signaling, biofilm cell death, eDNA release, and tobramycin-enhanced biofilm formation in *P. aeruginosa*. These specific adaptive mechanisms should be considered to improve treatment strategies against *P. aeruginosa* biofilm establishment in CF patients’ lungs.

## Introduction

Bacterial biofilm forms a highly structured community of cells that are attached to each other and/or a surface and are enclosed in a complex matrix of extracellular polymeric substances (EPS).^1,2^ Biofilms enable bacteria to colonize different environments and are prevalent in natural, industrial and medical environments. Importantly, biofilms have emerged as critical in chronic infections. The traits of bacteria within biofilms are distinct from those of their planktonic counterparts, which include an increased resistance to both biocide agents and antibiotics, the development of physical and social interactions, enhanced rate of gene exchange and selection for phenotypic variants.^3,4^ In many bacterial species, biofilm formation responds to a variety of environmental cues including nutritional availability, host-derived signals or, in some cases, to nonlethal concentrations of antibiotics.^5–8^ The process of biofilm development is coordinated by molecular pathways involving second-messenger signaling, cell-to-cell quorum sensing (QS) signaling, two-component systems and small noncoding RNAs (sRNAs).^6^ Interestingly, antibiotics at levels below the minimal inhibitory concentration (referred to hereafter as sub-MIC) have the ability to trigger the alteration of multiple physiological processes including biofilm formation, virulence, and gene expression, which can lead to bacterial genetic and phenotypic resistance.^9–12^ Sub-MICs of antibiotics with different chemical structures and modes of action induce biofilm formation in common clinical pathogens such as *Staphylococcus aureus, Enterococcus faecalis, Escherichia coli*, and *Pseudomonas aeruginosa*, among others.^5,11^

*P. aeruginosa* is a problematic Gram-negative pathogen representing a serious threat to individuals and public health. This opportunistic pathogen causes both acute and chronic infections that are strongly related to its planktonic and biofilm lifestyles, respectively. Within the lungs of cystic fibrosis (CF) individuals, biofilms are gradually formed by *P. aeruginosa* cells surrounded by a self-produced matrix of EPS such as polysaccharides, proteins, extracellular DNA (eDNA), metabolites, and siderophores.^2,13–15^ As a result of their ability to form biofilms and their high tolerance levels towards a broad spectrum of antimicrobials, *P. aeruginosa* chronic lung infections are almost impossible to eradicate.^13,16,17^ Tobramycin, an aminoglycoside antibiotic, is used in the treatment of *P. aeruginosa* infections.^18^ However, exposure to sub-MIC of this aminoglycoside^19–22^ and of other antibiotics such as quinolones^23^ and tetracycline^20,21^ enhances *P. aeruginosa* biofilm formation. Conversely, some other antibiotics such as polymyxin B, carbenicillin, and chloramphenicol, do not impact biofilm development.^19^

Based on microarray studies, tobramycin at the sub-MIC dose of 1 µg ml^−1^ led to altered expression of genes that are mainly involved in adaptation and protection processes in *P. aeruginosa* grown under planktonic conditions.^21^ Additionally, a recent study assessed the proteome response of planktonic cells of *P. aeruginosa* exposed to 0.1, 0.5, and 1 µg ml^−1^ sub-MIC of tobramycin.^24^ The authors identified higher abundances of multiple heat-shock proteins, proteases and proteins related to amino acid catabolic pathway. In contrast, they observed lower abundances of proteins associated with nucleotide metabolism, tricarboxylic acid (TCA), carbon metabolism and energy derivation, and electron transport activities. A small number of proteins were common to the proteomes produced at different sub-MICs of tobramycin while some proteins showed dose-dependent responses. It is worth to mention that aminoglycosides at sub-MICs can also induce other changes in *P. aeruginosa* physiology, including swimming and swarming motilities and the induction of the type VI secretion system (T6SS).^20,21^ Noteworthy, most of these studies have been conducted on bacteria grown under planktonic conditions. However, since bacteria are thought to adopt predominantly the biofilm lifestyle in nature and in infected host, it is crucial to perform studies on bacteria grown under sessile conditions.

In this context, we sought to elucidate adaptive mechanisms shaping the tobramycin-enhanced biofilm formation in *P. aeruginosa*. Remarkably, our observations support a potential adaptive mechanism in which the 4-hydroxy-2-alkylquinolines (HAQs) molecules and PrrF sRNAs are key players in eDNA release, presumably resulting from cell death which finally trigger changes in the biofilm architecture.

## Results

### Tobramycin exposure leads to changes in biofilm architecture, biovolume and thickness

**Fig. 1:**
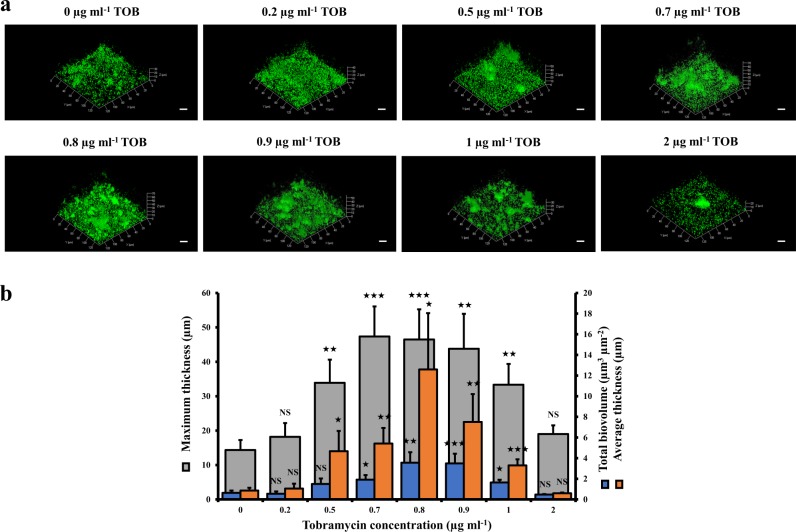
Effect of sub-MICs of tobramycin on biofilm formation by *P. aeruginosa*. **a** CLSM images of 24-h-old biofilms as a function of different concentrations of tobramycin. For each concentration, a 3D view along the *x*, *y* and *z* axes is displayed. Images show representative data from at least three independent biofilm assays. Scale bars = 20 µm. **b** COMSTAT image analyses were performed to determine maximum thicknesses (μm), average thicknesses (μm), and total biovolumes (μm^3^ μm^−2^). The error bars represent the standard error of the means (SEMs) and are the result of the analysis of three views of each of the three independent biological assays. Statistics were achieved by a two-tailed *t* test: ★★★, *P* = 0.0001 to 0.001; ★★, *P* = 0.001 to 0.01; ★, *P* = 0.01 to 0.05; NS (not significant), *P* ≥ 0.05

Previous studies showed enhanced *P. aeruginosa* biofilm formation upon exposure to tobramycin and other aminoglycosides by using colorimetric assays based on crystal violet staining.^19–21^ To observe the biofilm architectures and to quantify the biovolumes as well as the thicknesses of the biofilms, confocal laser scanning microscopy (CLSM) and COMSTAT image analyses were performed. First, we determined that the MIC of tobramycin for the *P. aeruginosa* wild-type H103 strain is 2 μg ml^−1^. Then, we grew *P. aeruginosa* H103 biofilms in glass bottom microplates under static conditions for 24 h in the presence of 0−2 μg ml^−1^ of tobramycin. Under our conditions, sub-MICs of tobramycin (0.5−1 μg ml^−1^) increased the presence of three-dimension (3D) structures in the biofilms (Fig. 1a). Consistently, at 0.7, 0.8, and 0.9 μg ml^−1^ tobramycin, the biofilm biovolumes, the maximum thicknesses, and the average thicknesses reached utmost significant increases compared to that of tobramycin-free biofilms (Fig. 1b). Thus, the concentration of 0.8 μg ml^−1^ of tobramycin was selected as the sub-MIC for all subsequent experiments.

### Extracellular DNA release and cell biofilm death increase in presence of tobramycin

**Fig. 2:**
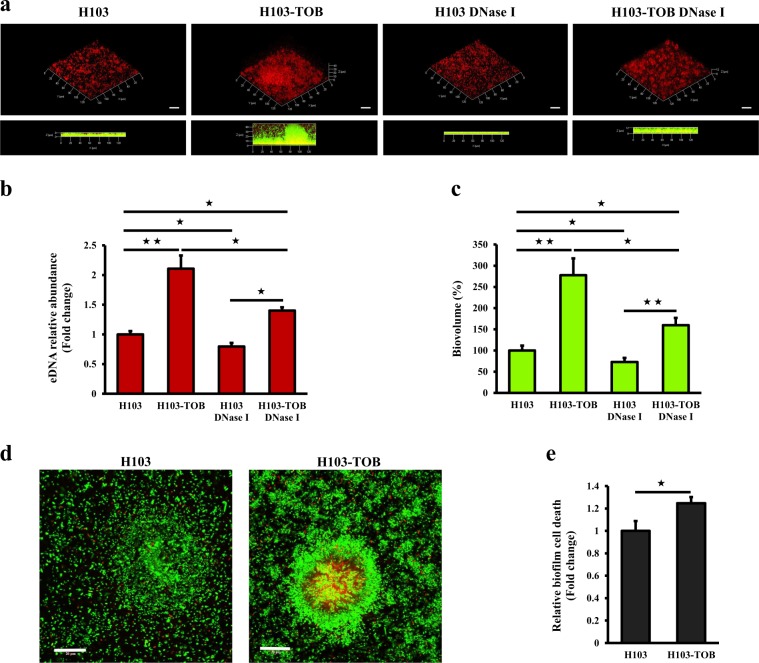
Sub-MIC of tobramycin leads to increased eDNA release and cell death in *P. aeruginosa* biofilm. **a** Representative CLSM 3D-top and side views of eDNA accumulation in 24-h-old *P. aeruginosa* biofilms exposed to tobramycin (0.8 μg ml^−1^) alone, DNase I (100 μg ml^−1^) alone or tobramycin and DNase I simultaneously, compared to untreated biofilms. Prior to image acquisition by CLSM, *P. aeruginosa* biofilm cells were labeled in green with SYTO 9 and the eDNA was stained in red with DDAO. Scale bars = 20 µm. CLSM images were analyzed using the COMSTAT software to quantify **b** the eDNA relative abundances relatively to biofilm biovolume values. Error bars represent standard error of the means (*n* = 3) and **c** the biofilm biovolumes. Error bars represent standard error of the means (*n* = 3). **d** CLSM micrographs of *P. aeruginosa* biofilms grown in the absence of tobramycin (left panel) and in the presence of drug (right panel) stained using the LIVE/DEAD^®^ *Bac*Light^TM^ Bacterial Viability Kit. Green fluorescent cells are viable, whereas red fluorescent cells have compromised cell membranes. Scale bars = 20 µm. **e** The cell death in biofilms was determined by COMSTAT images analyses. Values of nonviable biovolumes were normalized to total biovolumes. Error bars represent standard error of the means (*n* = 3). Statistics were achieved by a two-tailed *t* test: ★★, *P* = 0.001 to 0.01; ★, *P* = 0.01 to 0.05

We then asked whether eDNA, a major structural component of the *P. aeruginosa* biofilm matrix,^14,15,25^ contributes to the observed enhanced biofilm formation in response to tobramycin. CLSM and COMSTAT image analyses were used to evaluate the in situ eDNA level. The bacterial cells were labeled with the green fluorescent nucleic acid stain SYTO 9, and DDAO, a red fluorescent probe unable to cross the cell membranes, was used for eDNA staining. Figure 2a shows that the red labeling is more intense in a biofilm grown for 24 h in the presence of tobramycin than in a tobramycin-free biofilm, revealing a higher eDNA content in the first condition. Moreover, the red fluorescence was visible at the periphery of 3D structures in the presence of tobramycin, showing that eDNA might be involved, at least partly, in modifying the biofilm architecture in response to tobramycin. In the absence of tobramycin, a yellow coloration due to the superposition of the green (bacteria) and red (eDNA) fluorescence was observed, suggesting that eDNA was mostly localized within the biofilm. COMSTAT analyses indicated a 2.1-fold relative increase in eDNA abundance when biofilms were exposed to sub-MIC of tobramycin (Fig. 2b). To assess the impact of eDNA on biofilm formation, biofilms were grown with tobramycin, DNase I (100 μg ml^−1^), or tobramycin and DNase I simultaneously, and were compared to untreated biofilms. The addition of DNase I at the onset of biofilm formation decreased the biovolume of 24-h-old tobramycin-free biofilms by 27% (Fig. 2c). Interestingly, when biofilms were formed simultaneously in presence of DNase I and tobramycin, the total biofilm biovolume was reduced by 42.4% compared to biofilm formed under tobramycin exposure without DNase I (Fig. 2c). Similarly, COMSTAT images analyses indicated that while the eDNA relative abundance in biofilms of H103 grown in the presence of DNase I was decreased by about 20.2%, it was reduced by 33.5% in biofilms grown with both DNase I and tobramycin compared to tobramycin-exposed biofilms without DNase I. These results indicate that eDNA release contributes to a certain extent to tobramycin-enhanced biofilm formation by *P. aeruginosa* H103. Moreover, we attempted to assess if the eDNA release occurs through cell lysis. To this end, cell death was evaluated using the Live/Dead staining kit (Fig. 2d). COMSTAT analyses of CLSM images revealed that cell death was significantly increased from 1 to 1.25-fold (by about 25%) in *P. aeruginosa* H103 biofilms grown in the presence of tobramycin compared to tobramycin-free biofilms (Fig. 2e). Altogether, these data indicate that tobramycin at sub-MIC induced eDNA release, most likely through cell lysis, thereby modifying the matrix composition, which might contribute at least in part to the increased biofilm formation.

### Proteomic profiling of tobramycin-enhanced biofilm formation suggests a complex adaptive physiology

**Fig. 3:**
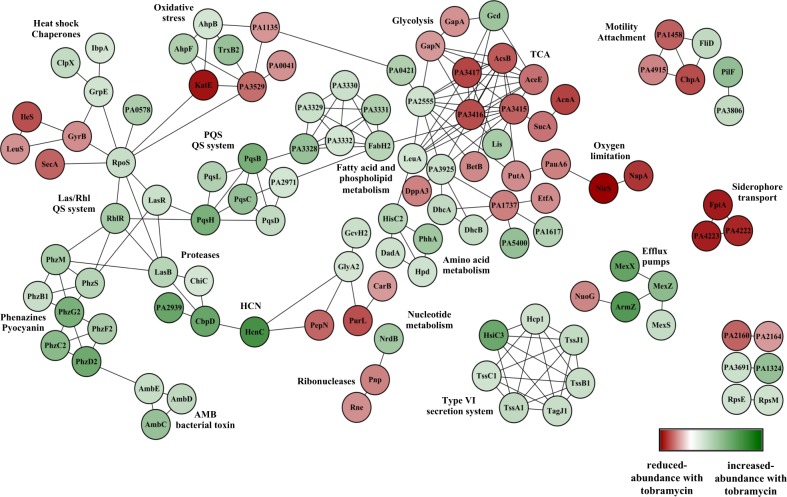
Protein−protein interaction network for highly differentially accumulated proteins in the *P. aeruginosa* biofilm cultures exposed to sub-MIC level of tobramycin. Nodes are colored according to the protein abundance (fold change). Nodes highlighted in green color correspond to the overaccumulated proteins whereas the nodes in red color represent the underaccumulated proteins. Edges indicate protein−protein interactions

To gain further insights into the tobramycin-enhanced biofilm formation potential adaptive mechanisms, the whole biofilm proteome was analyzed. The obtained results allowed the identification of 174 proteins with at least a twofold change in abundance in presence of sub-MIC tobramycin. Among these, 118 proteins showed increased abundances (Supplementary Table 1) while 56 were less abundant (Supplementary Table 2). An enrichment analysis with respect to PseudoCAP functional classes^26^ was performed for the reduced and increased-abundance proteins, respectively (Supplementary Fig. 1). The majority of enriched PseudoCAP functions for increased-abundance proteins belong to the categories of “secreted factors”, “adaptation and protection”, “antibiotic resistance and susceptibility”, “chaperones & heat shock proteins”, “amino acid biosynthesis and metabolism”, and “biosynthesis of cofactors, prosthetic groups and carriers” (Supplementary Fig. 1). By contrast, for reduced-abundance proteins, the PseudoCAP functional classes belong to “transcription, RNA processing, and degradation”, “energy metabolism”, “amino acid biosynthesis and metabolism”, and “carbon compound catabolism” (Supplementary Fig. 1). Next, a protein−protein interaction network was determined for proteins that were differentially produced in tobramycin-exposed biofilm. One hundred and ninety-one functional connections were inferred between 111 of the 174 proteins by selecting connections over a threshold of 0.7 of confidence combined score. The resulting string network was visualized within Cytoscape (version 3.2.1)^27^ (Fig. 3). The obtained results suggest a high number of interactions between the identified proteins which might be involved in the tobramycin-induced biofilm formation in *P. aeruginosa*. Interestingly, the proteome response to sub-MIC tobramycin treatment revealed increased levels of proteins associated with QS signaling networks, phenazine biosynthetic pathways and extracellular proteases (Supplementary Table 1; Fig. 3). On the other hand, the proteome analysis showed a decreased abundance of some proteins that are involved in central metabolism, including proteins associated with glycolysis, TCA cycle, and anaerobic metabolism (Supplementary Table 2; Fig. 3). Noticeably, the proteome of tobramycin-exposed *P. aeruginosa* biofilm showed reduced accumulation of ribonucleases RNase E (PA2976), RNase R (PA4937), and PNPase (PA4740) (Supplementary Table 2; Fig. 3), suggesting a possible reduced catabolism of RNAs. Overall, these proteomic data revealing alterations in the abundance of numerous proteins suggest complex adaptive mechanisms underlying tobramycin-increased biofilm development by *P. aeruginosa*.

### Tobramycin-enhanced biofilm formation is associated to production of QS molecules

**Fig. 4:**
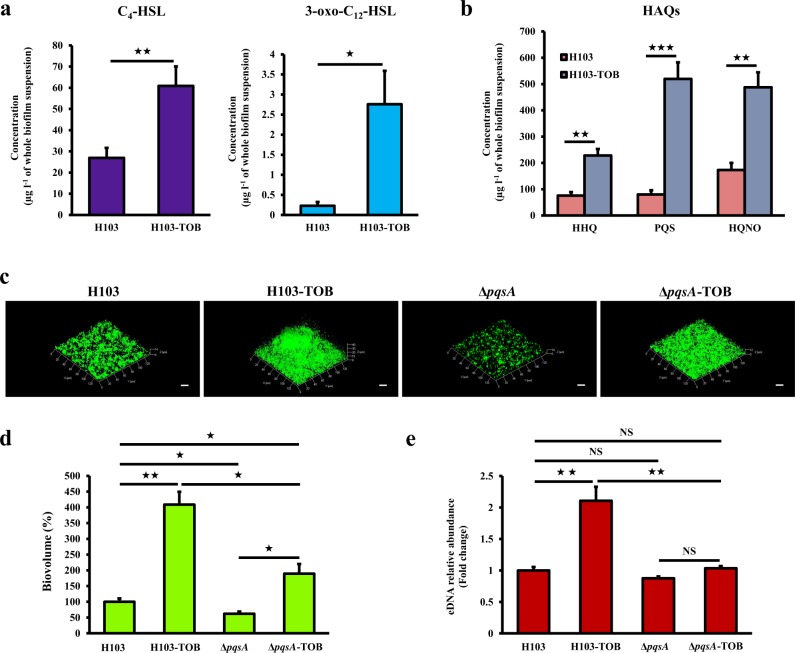
Tobramycin-enhanced biofilm formation is associated with increased production of QS molecules. **a** Quantification of the two main AHLs (C_4_-HSL and 3-oxo-C_12_-HSL) of Rhl and Las QS systems by LC-MS/MS analysis. Error bars represent standard error of the means (*n* ≥ 3). **b** Quantification of HAQ molecules (HHQ, PQS, and HQNO) by LC-MS/MS analysis. Error bars represent standard error of the means (*n* ≥ 3). **c** Representative CLSM 3D-top micrographs of 24-h-old biofilms of *P. aeruginosa* H103 and Δ*pqsA* mutant strains exposed to tobramycin (0.8 μg ml^−1^) compared to untreated biofilms. Scale bars = 20 µm. CLSM images were analyzed using COMSTAT software to quantify **d** biofilm biovolumes. Error bars represent standard error of the means (*n* = 3) and **e** eDNA relative abundances. Error bars represent standard error of the means (*n* = 3). eDNA values were normalized to biofilm biovolumes. Asterisks indicate a significant difference as determined by a two-tailed *t* test: ★★★, *P* = 0.0001 to 0.001; ★★, *P* = 0.001 to 0.01; ★, *P* = 0.01 to 0.05; NS (not significant), *P* ≥ 0.05

Since QS signaling molecules have been reported to be involved in eDNA release via subpopulation cell lysis,^28^ the ability of tobramycin to increase the production of QS-related molecules in colony biofilms of H103 strain was determined using LC-MS/MS quantification. As shown in Fig. 4a, tobramycin significantly increased the production of the two main *P. aeruginosa N*-acyl-homoserine lactones (AHLs), *N*-(3-oxododecanoyl)-l-homoserine lactone (3-oxo-C_12_-HSL), and *N*-butanoyl-l-homoserine lactone (C_4_-HSL). Furthermore, the major molecules from the HAQs family, namely 3,4-dihydroxy-2-heptylquinoline (termed the *Pseudomonas* quinolone signal (PQS)), its precursor 4-hydroxy-2-heptylquinoline (HHQ) and 4-hydroxy-2-heptylquinoline *N*-oxide (HQNO) were all significantly more abundant upon tobramycin exposure (Fig. 4b). Accordingly, the proteomic analyses revealed an enhanced production of PqsB (PA0997), PqsC (PA0998), and PqsD (PA0999) that are involved in HHQ biosynthesis (Supplementary Table 1; Fig. 3). This was also the case for both PqsH (PA2587), responsible for the conversion of HHQ into PQS,^29,30^ and PqsL (PA4190), which is required for HQNO biosynthesis^31^ (Supplementary Table 1; Fig. 3). These results provide evidence that sub-MIC of tobramycin induces the production of QS-related molecules, especially PQS and HQNO that might be involved in eDNA release and/or cell lysis, leading partly to the observed tobramycin-increased biofilm formation in *P. aeruginosa*. To support this hypothesis, a *pqs* mutant strain (Δ*pqsA*) was constructed to determine the impact of HAQ molecules on biofilm formation and eDNA release in tobramycin-exposed biofilms (Fig. 4c). As expected, HAQs levels determined by LC/MS-MS were shown to be abolished in Δ*pqsA* biofilm exposed or not to tobramycin compared to HAQs levels of H103 wild-type strain (Supplementary Table 3). The Δ*pqsA* mutant displayed reduced biofilm biovolume (by about 38%) compared to H103. The presence of tobramycin in Δ*pqsA* biofilm cultures increased the biovolume compared to Δ*pqsA* tobramycin-free biofilms as in the case of H103 treated or not with tobramycin but in a minor rise proportion (Fig. 4d). Noticeably, the biofilm biovolume of Δ*pqsA* biofilms grown in presence of sub-MIC of tobramycin was significantly reduced (53.6%) when compared to the biovolume of H103 biofilms grown in the same conditions. By contrast, the eDNA relative abundance did not increase in Δ*pqsA* biofilms in presence of tobramycin compared to biofilms of the same strain without tobramycin, on the opposite to the tobramycin-induced eDNA increase seen in H103 biofilms (Fig. 4e). The relative eDNA abundances were thus not significantly different in tobramycin-free H103 biofilms and Δ*pqsA* biofilms with or without antibiotic, and were only higher in H103 biofilms with tobramycin (Fig. 4e). Taken together, these data indicate that whereas the biofilm biovolume enhancement could still occur in response to sub-MIC of tobramycin in Δ*pqsA* biofilms, at least a part of the increase of eDNA release appears to be HAQ-dependent.

### PrrF sRNAs promote tobramycin-enhanced biofilm formation

**Fig. 5:**
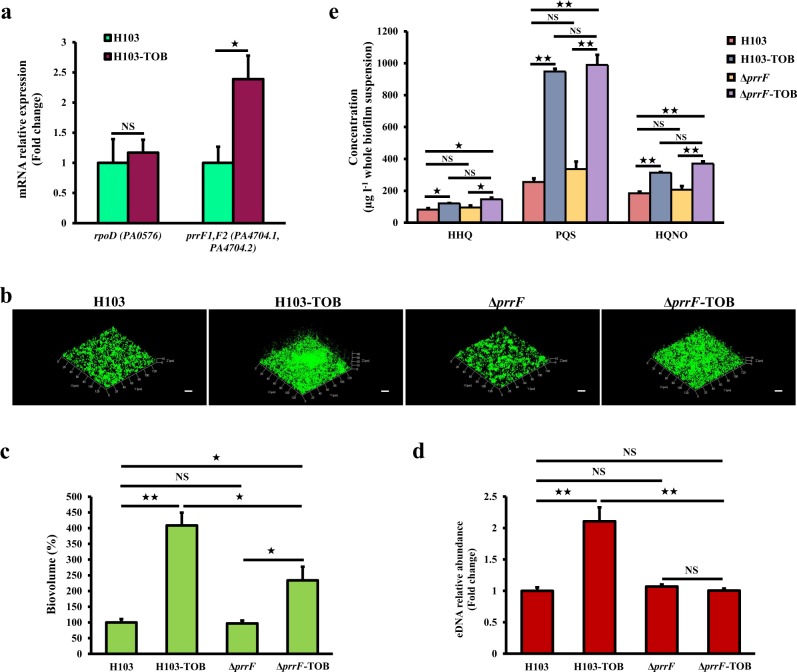
Role of PrrF1/F2 sRNAs in tobramycin-enhanced biofilm formation. **a** Relative *prrF* mRNA levels in *P. aeruginosa* biofilms exposed to tobramycin (brown bars) compared to the relative expression of mRNA levels in the control condition (green bars), after 24 h of growth. Quantifications have been obtained from at least three independent experiments and *rpoD* was used as a control housekeeping gene. Error bars represent standard error of the means (*n* = 3). **b** Representative CLSM 3D micrographs of 24-h-old biofilms *P. aeruginosa* H103 and Δ*prrF* mutant strains exposed to tobramycin (0.8 μg ml^−1^) compared to untreated biofilms. Scale bars = 20 µm. CLSM images were analyzed using COMSTAT software to quantify **c** biofilm biovolumes. Error bars represent standard error of the means (*n* = 3) and **d** eDNA relative abundances. eDNA values are normalized to biofilm biomass. Error bars represent standard error of the means (*n* = 3). **e** HAQs levels of the Δ*prrF* mutant and its isogenic parent strain H103 grown with or without tobramycin supplementation, as determined by LC-MS/MS. Error bars represent standard error of the means (*n* = 3). Statistics were achieved by a two-tailed *t* test: ★★, *P* = 0.001 to 0.01; ★, *P* = 0.01 to 0.05; NS (not significant), *P* ≥ 0.05

A previous study reported that biofilm formation in the *prrF* mutant is not induced under sub-MIC of tobramycin.^32^ Thus, the effect of tobramycin was assessed on PrrF sRNAs expression using RT-qPCR. Interestingly, the expression of PrrF sRNAs was significantly increased by 2.4-fold (Fig. 5a), suggesting their involvement in the tobramycin-enhanced biofilm formation in *P. aeruginosa*. To further validate this hypothesis, biofilm formation and eDNA release were evaluated in a Δ*prrF* mutant under tobramycin exposure (Fig. 5b). As seen in Fig. 5c, no significant difference was observed between the biofilm biovolume of *P. aeruginosa* Δ*prrF* mutant and H103 strains in the absence of antibiotic. Remarkably, sub-MIC of tobramycin-enhanced biofilm biovolume in Δ*prrF* mutant compared to Δ*prrF* tobramycin-free biofilm as in the case of the biofilm formation of H103 grown without or with tobramycin but in a minor rise proportion (Fig. 5c). However, tobramycin-treated biofilm of Δ*prrF* mutant did not show increased relative abundance of eDNA (Fig. 5d). Interestingly, HAQs quantification by LC-MS/MS showed that deletion of PrrF sRNAs had no significant effect on the production levels of HHQ, PQS, and HQNO when compared to H103 strain grown in the same conditions (Fig. 5e). Moreover, the HAQs levels in the exposed-tobramycin biofilm of Δ*prrF* mutant were increased to levels at those of the H103 biofilm treated with tobramycin (Fig. 5e). Altogether, these results indicate a link between PrrF sRNAs and eDNA release probably mediated through an HAQ-independent mechanism in response to sub-MIC of tobramycin.

### Effect of tobramycin on iron uptake systems and oxidative stress response in biofilm

**Fig. 6:**
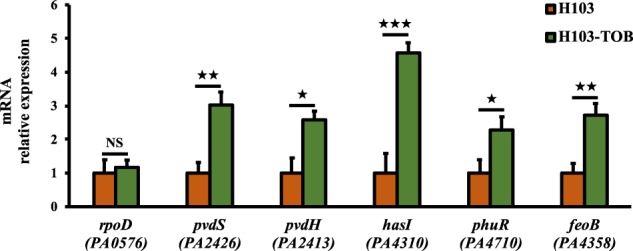
Sub-MIC of tobramycin enhance iron/heme uptake strategies in *P. aeruginosa* biofilm cultures. Relative *pvdS*, *pvdH*, *hasI*, *phuR*, and *feoB* mRNA levels in *P. aeruginosa* biofilm cultures exposed to sub-MIC of tobramycin (green bars) compared to the relative mRNA levels in the control condition (brown bars), after 24 h of growth. Quantifications have been obtained from at least three independent experiments and *rpoD* was used as a control housekeeping gene. Error bars represent standard error of the means (*n* = 3). Statistics were achieved by a two-tailed *t* test: ★★★, *P* = 0.0001 to 0.001; ★★, *P* = 0.001 to 0.01; ★, *P* = 0.01 to 0.05

The PrrF1 and PrrF2 sRNAs are key elements of *P. aeruginosa* iron homeostasis^32^ and previous studies demonstrated the requirement of iron for induction of biofilm formation in *P. aeruginosa* by sub-MIC of tobramycin.^33^ Therefore, the effect of tobramycin was explored on the expression of iron and heme acquisition systems. RT-qPCR analyses indicated increased expression of *pvdS* encoding the PvdS extracytoplasmic function sigma factor (ECFσ)^34^ and the *pvdH* biosynthetic gene by about threefold in response to tobramycin (Fig. 6). Similarly, expression of *hasI* and *phuR*, encoding the HasI ECFσ for the Has-dependent heme uptake system and heme/hemoglobin outer membrane receptor PhuR increased in the presence of tobramycin, reflecting the activity of the heme acquisition (Has) and the heme uptake (Phu) systems (Fig. 6). Finally, we assayed the Feo system for the uptake of Fe^2+^ and showed enhanced expression of *feoB* encoding the inner membrane permease FeoB (Fig. 6). The enhancement of these iron uptake systems in response to tobramycin reveals a high demand of iron that might lead to oxidative damages. Interestingly, proteomic data analysis showed that the two alkyl hydroperoxide reductases AhpF (PA0140) and AhpB (PA0848) were significantly more abundant in tobramycin-exposed biofilm. Additionally, the protein TrxB (PA0849), a thioredoxin reductase, which protects protein disulfide bonds from oxidation was also induced (Supplementary Table 1; Fig. 3). Collectively, these data suggest a contribution of iron and oxidative stress responses into the tobramycin-enhanced biofilm formation in *P. aeruginosa* that could be mediated by the PrrF sRNAs.

## Discussion

Bacteria have the ability to adapt to numerous environmental stresses by setting up different biological responses such as the enhancement of biofilm formation, notably upon exposure to low antibiotic concentrations.^12,13^ Through CLSM observations, whole biofilm proteome analysis, gene expression RT-qPCR assays, and phenotypic approaches, we present herein further insights into the adaptation mechanisms leading to increased biofilm formation in *P. aeruginosa* in response to sub-MIC of tobramycin. The effect of sub-MIC of tobramycin on increased biofilm development in *P. aeruginosa* was corroborated^19–21^ and the present work demonstrates modifications in the biofilm architecture. Remarkably, tobramycin exposure results in biofilm matrix modifications by increasing eDNA abundance levels, which in turn favors the built up of 3D structures^15^ leading to elevated biofilm biovolume and thickness, as confirmed by DNase I treatment. This result mirrors those of previous studies that have shown increased matricial extracellular nucleic acids amounts in *Enterococcus faecalis* and *Staphylococcus aureus* in response to sub-MICs of ampicillin and methicillin, respectively.^35,36^

eDNA was previously described to play a structural role in biofilm formation, to bind and shield biofilms from aminoglycosides, and to induce antimicrobial peptide resistance mechanisms in *P. aeruginosa*.^37–40^ The observed rise in eDNA release is presumably a consequence of elevated cell biofilm death that seems to be mediated by a QS-dependent mechanism. Accordingly, data of the current study demonstrate increased production levels of HAQ molecules (HHQ, PQS, and HQNO) in *P. aeruginosa* H103 strain and decreased eDNA release in Δ*pqsA* in response to sub-MIC of tobramycin. This finding is in agreement with a previous study which showed that HAQs play a key role in eDNA release, occurring as a result of biofilm subpopulations lysis.^28^ However, given that Δ*pqsA* biofilm biovolume decreased, we have been unable to show differences in terms of eDNA release between *pqsA* mutant and H103 strains in the absence of tobramycin. This result was unexpected and suggests that an alternative potential eDNA-independent mechanism might be involved. Besides, HQNO is a well-known molecule that can act as a cytochrome inhibitor of the respiratory chain,^41^ inducing thus the production of reactive oxygen species (ROS), which are involved in membrane damages resulting in cell autolysis and eDNA release.^42^ We found here also that proteins related to phenazine biosynthesis and several extracellular proteases triggered through the QS system are enhanced in presence of sub-MIC tobramycin. Previous findings demonstrated that pyocyanin enhances the matrix stability by interacting with eDNA, thus enabling the strength and development of the biofilm.^43,44^ Moreover, the current study demonstrated significant higher production levels of the two AHL signal molecules, 3-oxo-C_12_-HSL and C_4_-HSL, produced by the Las and Rhl QS systems, respectively, that are involved in *P. aeruginosa* biofilm development.^13,45^ The induction of the Las system under tobramycin exposure may trigger the observed increased accumulation of proteins related to HAQ biosynthesis. Altogether these data suggest a key role for the HAQ, including in signaling, and probably also ROS in the release of eDNA leading to increased biofilm development in response to sub-MIC of tobramycin.

Previous studies reported that PQS triggers iron-starvation response in *P. aeruginosa* by its ability to chelate ferric iron (Fe^3+^) and functions as an iron trap associated with the outer membrane.^46,47^ In fact, several iron acquisition systems are overexpressed in *P. aeruginosa* biofilm under tobramycin exposure. This study demonstrates higher expression levels of genes involved in the biosynthesis of pyoverdine siderophore, which is of importance for biofilm formation.^48,49^ The two heme uptake systems Has and Phu and the ferrous iron uptake Feo system are also increased. Remarkably, phenazine-1-carboxylic acid, that can reduce ferric iron to its ferrous oxidation state, promotes biofilm formation through the Feo system.^50^ Thus, these results reveal a contribution of iron uptake strategies in tobramycin-enhanced biofilm formation in *P. aeruginosa*. These findings are in line with a previous study that demonstrated the role of iron in the induction of biofilm formation in *P. aeruginosa* by sub-MIC of tobramycin.^33^ While the potential of iron to stimulate oxidative stress, the proteomic data of this study showed a rise of the AhpF, AhpB-TrxB2 proteins responsible for an antioxidant mechanism activity^51^ that would help limiting some adverse effects of ROS. In addition, the PqsR (MvfR) transcriptional regulator activated by PQS modulates the expression of genes involved in the oxidative stress response.^51^

Besides, the iron-starvation response revealed by gene expression data seems to be involved in the increased expression of regulatory PrrF sRNAs that are known to regulate several genes involved in protection from oxidative stress and iron storage.^32,52^ Remarkably, an important finding of this study was that biofilms formed by Δ*prrF* in presence of tobramycin contain less eDNA than H103 strain biofilm formed under the same condition, therefore following a similar trend as the Δ*pqsA* mutant when compared to *P. aeruginosa* H103 strain under tobramycin exposure. However, while PrrF sRNAs were shown previously to modulate HAQs production under planktonic conditions,^53,54^ our study that was conducted in biofilms showed that Δ*prrF* mutant produced HAQs levels similar to those of the wild-type strain grown with or without tobramycin. These data suggest that HAQs production modulation by PrrF sRNAs might not play an important role in H103 strain and studied conditions and point out to the complex regulation of HAQs production. In summary, these data suggest that regulatory PrrF sRNAs are key players mediating eDNA release through possibly another potential HAQ-independent mechanism leading to tobramycin-increased biofilm formation. Furthermore, the whole proteome data show decreased abundances of RNases including RNaseE, RNaseR, and PNPase in the presence of sub-MIC of tobramycin that appears to explain the increased expression of sRNAs. Recent studies indicate that RNases greatly affect biofilm formation by causing sRNAs decay in microorganisms such as *P. aeruginosa*, *Escherichia coli* and *Salmonella* Typhimurium.^55–57^

In addition, numerous metabolic pathways were affected in the whole biofilm proteome exposed to tobramycin. Especially, proteins related to glycolysis, TCA, and denitrification pathways were under-produced, suggesting a metabolic adaptation under biofilm growth in the presence of tobramycin. Interestingly, a recent study reported the functional enrichment of proteins related to TCA, carbon metabolism and energy derivation, and electron transport activities, which were under-accumulated in the presence of tobramycin under planktonic conditions.^24^ *P. aeruginosa* was demonstrated to use PQS to downregulate genes involved in denitrification in planktonic conditions,^58^ indicating a link between QS and energy metabolism. Recently, AHL-mediated QS was shown to alter TCA cycle intermediates, and fatty acid and amino acid metabolism during stationary phase.^59^ Amino acid metabolism (as carnitine and lysine) was also affected upon tobramycin exposure in our study according to previous data observed when *P. aeruginosa* was grown in planktonic conditions.^24^ Moreover, and in line with our results, the enhanced catabolism of the amino acids arginine, phenylalanine and tyrosine was reported.^60^

Overall, we suggest herein new potential adaptive mechanisms on how sublethal concentrations of tobramycin lead to increased biofilm formation in *P. aeruginosa*. Importantly, the release of eDNA might explain, at least in part, the enhancement of biofilm formation and point to the fundamental role of the biofilm matrix. HAQs and regulatory PrrF sRNAs appears to be key players in the eDNA release since no effect of tobramycin was observed on eDNA release in Δ*pqsA* and Δ*prrF* mutants. However, there was still an impact of tobramycin on Δ*pqsA* and Δ*prrF* biofilm formation suggesting that alternative mechanism(s) may possibly be involved. In addition, it is apparent that the decreased eDNA release observed in Δ*pqsA* and Δ*prrF* mutants occur through distinct mechanisms and we cannot disregard the importance of the other discussed adaptive mechanisms which deserve further investigations.

## Methods

### Bacterial strains, growth conditions and antibiotics

The *P. aeruginosa* H103 strain and derivatives are listed in Supplementary Table 4. *P. aeruginosa* H103 biofilms were grown on membrane filters (25 mm diameter, 0.2 µm pore size, Whatman) placed over the surface of freshly prepared LB agar plates supplemented or not with the required concentration of tobramycin (Sigma-Aldrich). To this end, overnight planktonic cultures grown aerobically at 37 °C in LB broth on a rotary shaker (180 r.p.m.) were diluted to an OD at 580 nm of 1 and 100 µl spotted on the membrane. The bacteria were incubated at 37 °C for 24 h on static conditions. The antibiotics stock solutions used in this study were sterilized by filtration through 0.22-µm filters, aliquoted into daily-use volumes and kept at −20 °C.

### Sensitivity of *P. aeruginosa* to tobramycin

ETEST^®^ tobramycin gradient strip (0.016−256 µg ml^−1^; Biomérieux) was used to assess the MIC assay for *P. aeruginosa* strain H103 according to the manufacturer’s instructions. An inoculum containing a concentration of bacteria that approximates the 0.5 McFarland turbidity standard was used. Antibiotic sensitivity was read after 24 h incubation at 37 °C.

### Construction of the Δ*pqsA* and Δ*prrF* mutant strains

The *pqsA* and *prrF* mutant strains were constructed by following the procedure described by Quénée et al.^61^ Briefly, the *pqsA* and *prrF*-flanking regions were PCR amplified using the primer pairs listed in Supplementary Table 5. PCR products *pqsA*- or *prrF*-upstream and *pqsA*- or *prrF*-downstream were digested by *Sac*I*/Xba*I or *Eco*RI*/Xba*I and *Xba*I*/Hin*dIII, respectively, and cloned by a three-way ligation into the suicide vector pEX100Tlink opened by *Sac*I and *Hin*dIII or *Eco*RI and *Hin*dIII. These cloning strategies resulted in the construction of pEX:Δ*pqsA* and pEX:Δ*prrF* plasmids, respectively. The *lox-aacC1-lox* cassette encoding gentamicin (Gm) resistance was excised from pUCGm*lox*^61^ using *Xba*I and was subcloned into the unique *Xba*I site of pEX:Δ*pqsA* and pEX:Δ*prrF*. The resulting pEX:Δ*pqsA*:Gm*lox* and pEX:Δ*prrF*:Gm*lox* plasmids were then introduced into the *E. coli* donor/helper strain S17.1 and transferred by conjugation into *P. aeruginosa* H103. Gm-resistant colonies grown on Pseudomonas isolation agar (PIA) plates were counter-selected on 5% sucrose LB agar plates, and the double recombinants were selected for their Gm resistance and carbenicillin (Cb) sensitivity. Finally, the *aacC1* gene conferring Gm resistance was excised by the Cre recombinase encoded by pCM157.^62^ The resulting Δ*pqsA* and Δ*prrF* mutant strains were checked by PCR using primer pairs pqsA-*Sac*I-F/pqsA-*Hin*dIII-R and prrF-*Eco*RI-F/prrF-*Hin*dIII-R, respectively (Supplementary Table 5), and the resulting fragments were verified by DNA sequencing (Sanger sequencing services, Genewiz).

### Confocal laser scanning microscopy (CLSM)

*P. aeruginosa* H103, Δ*pqsA*, and Δ*prrF* biofilms were grown in LB medium supplemented or not with the required concentration of tobramycin on a 24-well glass-bottomed microplates for 24 h at 37 °C in static conditions. Biofilm formation in the presence of DNase I from bovine pancreas (Sigma-Aldrich) was performed by supplementing LB medium with DNase I at 100 µg ml^−1^.^63^ Prior to image acquisition, biofilm cells and eDNA matrix component were stained with fluorescent dyes and observed by CLSM. Biofilm cells were stained by adding 5 µM of SYTO^®^ 9 green fluorescent nucleic acid stain (Invitrogen, Thermo Fisher Scientific; excitation at 488 nm and emission from 500 to 550 nm) or 5 µM of SYTO^®^ 62 red fluorescent nucleic acid stain (Molecular Probes, Life Technologies; excitation at 652 nm and emission at 676 nm) prepared in sterile physiological solution (0.9% NaCl), incubated at room temperature for 15 min in the dark and then washed twice with sterile physiological solution (0.9% NaCl).

The eDNA biofilm-component matrix was stained using 1 µM of 7-hydroxy-9H-(1,3-dichloro-9,9-dimethylacridin-2-one) (DDAO), a red fluorescent probe (Molecular Probes, *life*; excitation at 663 nm and emission at 660 nm).

The Live/Dead fluorescent staining was performed using the LIVE/DEAD^®^ *BacLight*^TM^ Bacterial Viability Kit (Thermo Fisher Scientific). The cells were labeled with a mixture (v/v) of component A (SYTO 9 dye, 1.67 mM/propidium iodide, 1.67 mM) and component B (SYTO 9 dye, 1.67 mM/propidium iodide, 18.3 mM) according to the manufacturer’s recommendations.

The CLSM observations were carried out with a Zeiss LSM710 microscope (Carl Zeiss Microscopy) using a ×63 oil immersion objective. Images were taken every micrometer throughout the whole biofilm depth. For visualization and processing of 3D images, the Zen 2.1 SP1 zen software (https://www.zeiss.com/microscopy/int/downloads/zen.html) (Carl Zeiss Microscopy) was used. The thicknesses (μm) and biovolumes (μm^3^ μm^−2^) of the biofilms were measured using the COMSTAT2 software (http://www.imageanalysis.dk/).^64^ At least three image stacks from each of three independent experiments were used for each analysis.

### Reverse transcription-quantitative PCR analysis (RT-qPCR)

Total RNAs from three independent biofilm cultures were isolated by the hot acid-phenol method,^65^ followed by treatment with Turbo DNA-*free*^TM^ kit (Invitrogen) according to the manufacturer’s protocol. Synthesis of cDNAs and RT-qPCR was achieved as previously described,^66^ using the oligonucleotides listed in Supplementary Table 3. The mRNAs and sRNAs levels were calculated by comparing the threshold cycles (Ct) of target genes with those of control sample groups and the relative quantification was measured using the 2^−ΔΔCt^ method^67^ using DataAssist^TM^ software (Applied Biosystems).

### LTQ-orbitrap analysis

The sample preparation, protein digestion, tandem mass spectrometry and whole proteome analysis was performed as previously described.^68^ All experiments were performed on an LTQ-Orbitrap Elite (Thermo Scientific) coupled to an Easy nLC II system (Thermo Scientific). One microliter of sample was injected onto an enrichment column (C18 PepMap100, Thermo Scientific). The separation was performed with an analytical column needle (NTCC-360/100-5-153, NikkyoTechnos, Japan). The mobile phase consisted of H_2_O/0.1 % formic acid (FA) (buffer A) and CH_3_CN/FA 0.1 % (buffer B). Tryptic peptides were eluted at a flow rate of 300 nl/min using a three-step linear gradient: from 2 to 40% B over 75 min, from 40 to 80% B in 4 min and 11 min at 80% B. The mass spectrometer was operated in positive ionization mode with capillary voltage and source temperature set at 1.5 kV and 275 °C, respectively. The samples were analyzed using CID (collision induced dissociation) method. The first scan (MS spectra) was recorded in the Orbitrap analyzer (*R* = 60,000) with the mass range *m/z* 400–1800. Then, the 20 most intense ions were selected for MS^2^ experiments. Singly charged species were excluded for MS^2^ experiments. Dynamic exclusion of already fragmented precursor ions was applied for 30 s, with a repeat count of 1, a repeat duration of 30 s and an exclusion mass width of ±10 ppm. Fragmentation occurred in the linear ion trap analyzer with collision energy of 35%. All measurements in the Orbitrap analyzer were performed with on-the-fly internal recalibration (lock mass) at *m/z* 445.12002 (polydimethylcyclosiloxane). After MS analysis, raw data were imported in Progenesis LC-MS software (Nonlinear Dynamics). For comparison, one sample was set as a reference and the retention times of all other samples within the experiment were aligned. After alignment and normalization, statistical analysis was performed for one-way analysis of variance (ANOVA) calculations. Peptide features presenting a *p* value and a *q* value less than 0.05, and a power greater than 0.8 were retained. MS/MS spectra from selected peptides were exported for peptide identification with Mascot (Matrix Science) against the database restricted to *P. aeruginosa* PAO1 (http://www.pseudomonas.com).^26^ Database searches were performed with the following parameters: 1 missed trypsin cleavage site allowed; variable modifications: carbamidomethylation of cysteine and oxidation of methionine. Peptides with scores above 20 were imported into Progenesis. For each condition, the total cumulative abundance of the protein was calculated by summing the abundances of peptides. Proteins identified with less than two peptides were discarded. Only the proteins that varied by twofold in these average normalized abundances between growth conditions were retained. Expression data for all significantly differentially produced proteins are available in Supplementary Tables 1 and 2.

### Functional enrichment of proteomic data

The enrichment factor (EF) was calculated using the following formula: EF = (number of specific PseudoCAP classes detected/number of all PseudoCAP classes detected)/ (number of specific PseudoCAP classes annotated/number of all PseudoCAP classes annotated). Functional categories displaying an EF ≥ 1.5 are defined as overrepresented in the functional proteomic profiling of the tobramycin-exposed biofilm (Supplementary Fig. 1).

### *P. aeruginosa* whole biofilm Protein−Protein Interaction Network (PPIN) inference

*P. aeruginosa* PAO1 Protein−Protein Interaction Network (PPIN) was retrieved from the STRING database (http://string-db.org/).^69^ One hundred and ninety-one functional connections were inferred between 111 proteins of the 174 proteins by selecting connections over a threshold of 0.7 of confidence combined score. The 63 proteins without any connection to other proteins in the network were removed. The resulting string network was visualized within Cytoscape (version 3.2.1) (http://www.cytoscape.org).^27^

### Extraction and quantification of AHLs and HAQs

Colony biofilms of H103 strain and its derivative mutants exposed or not to tobramycin were resuspended in 0.9% NaCl (three-colony biofilms per 10 ml of 0.9% NaCl). Biofilm suspensions were vortexed for 2 min and the AHL and HAQ molecules were extracted following the technique described in a previous study.^70^ AHLs and HAQs were quantified by liquid chromatography coupled to mass spectrometry (LC-MS/MS).^29,71^ The obtained data were normalized to OD of biofilm suspensions. AHL standards were obtained from Sigma [*N*-butanoyl-l-homoserine lactone (C_4_-HSL) and *N*-3-oxododecanoyl-l-homoserine lactone (3-oxo-C_12_-HSL)].

### Statistical analysis

Statistical significance was evaluated using Prism GraphPad online tool (https://www.graphpad.com/quickcalcs/ttest1/). The data were statistically analyzed using unpaired (two sample) two-tailed *t* test to calculate *p* values. The mean with standard error of the mean (SEM) were calculated and plotted.

### Reporting summary

Further information on experimental design is available in the Nature Research Reporting Summary linked to this paper.

## Supplementary information

Reporting Summary Checklist

Supplementary Information

## Data Availability

The authors declare that all relevant data supporting the findings of the study are available in this article and its Supplementary Information files, or from the corresponding author upon request.

## Supplementary information

**Supplementary information** accompanies the paper on the *npj Biofilms and Microbiomes* website (10.1038/s41522-019-0088-3).
