# Supplementary material for: Extracellular DNA release, quorum sensing, and PrrF1/F2 small RNAs are key players in Pseudomonas aeruginosa tobramycin-enhanced biofilm formation
Source: NPJ Biofilms Microbiomes. 2019 May 23;5:15. doi: 10.1038/s41522-019-0088-3 (PMC6533273; doi:10.1038/s41522-019-0088-3)
Supplement: Supplementary file 2 — Supplementary Information [file 41522_2019_88_MOESM2_ESM.pdf]

Supplementary Information

Extracellular DNA release, quorum sensing, and PrrF1/F2 small RNAs are key players in *Pseudomonas aeruginosa* tobramycin-enhanced biofilm formation

Ali Tahrioui[1#], Rachel Duchesne[1], Emeline Bouffartigues[1], Sophie Rodrigues[1], Olivier Maillot[1], Damien Tortuel[1], Julie Hardouin[2], Laure Taupin[3], Marie-Christine Groleau[4], Alain Dufour[3], Eric Déziel[4], Gerald Brenner-Weiss[5], Marc Feuilloley[1], Nicole Orange[1], Olivier Lesouhaitier[1], Pierre Cornelis[1] and Sylvie Chevalier[1]

[1]Normandie Université, Université de Rouen Normandie, Laboratoire de Microbiologie Signaux et Microenvironnement, LMSM EA4312, Évreux, France

[2]Normandie Université, Université de Rouen Normandie, Laboratoire Polymères Biopolymères Surfaces, PBS, UMR 6270 CNRS, Mont Saint Aignan, France

[3]Université de Bretagne-Sud, Laboratoire de Biotechnologie et Chimie Marines, LBCM EA3884, Institut Universitaire Européen de la Mer, Lorient, France

[4]INRS-Institut Armand-Frappier, Laval, Québec, Canada

[5]Institute of Functional Interfaces, Karlsruhe Institute of Technology, Karlsruhe, Germany

Supplementary Figure 1

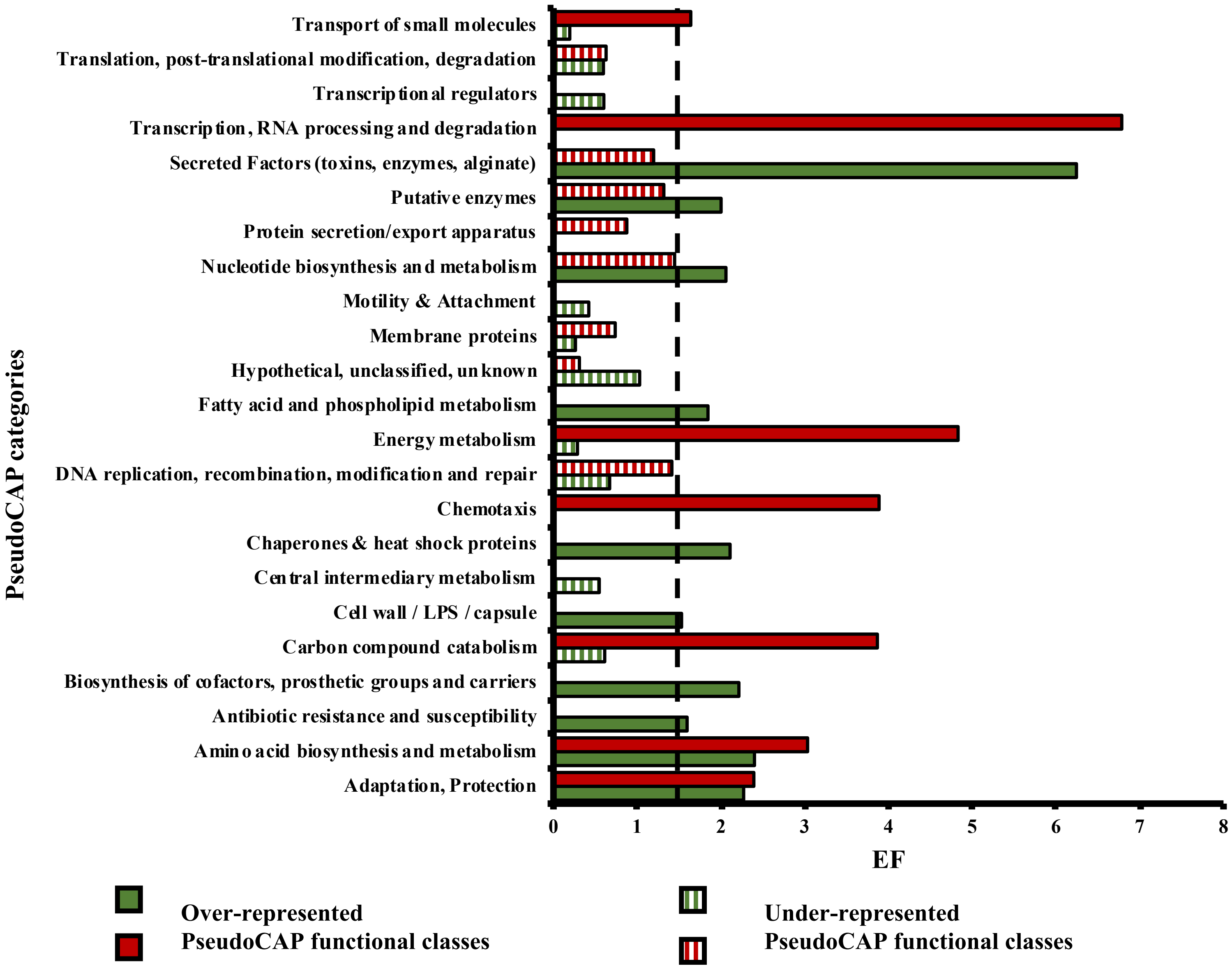

Supplementary Figure 1 Functional classification of proteins that are over-represented (red and green bars) and under-represented (red and green striped-bars) in *P. aeruginosa* biofilm cultures exposed to sub-MIC of tobramycin. The PseudoCAP annotation (www.pseudomonas.com) was used to categorize the members of the significantly differentially abundant proteins, and enrichment of specific classes of proteins relative to their distribution in PAO1 is displayed as the enrichment factor (EF).

Supplementary Table 1 List of most differentially abundant proteins in tobramycin-enhanced biofilm formation of *P. aeruginosa*

Locus Tag	Fold Change	Name	Product Description	PseudoCAP Class category
PA0080	2.7	TssJ1	TssJ1	Protein secretion/export apparatus; Hypothetical, unclassified, unknown
PA0082	2.8	TssA1	TssA1	Protein secretion/export apparatus; Hypothetical, unclassified, unknown
PA0083	2.5	TssB1	TssB1	Protein secretion/export apparatus; Hypothetical, unclassified, unknown
PA0084	2.1	TssC1	TssC1	Protein secretion/export apparatus; Hypothetical, unclassified, unknown
PA0085	2.3	Hcp1	Hcp1	Secreted Factors (toxins, enzymes, alginate)
PA0086	2.4	TagJ1	TagJ1	Protein secretion/export apparatus; Hypothetical, unclassified, unknown
PA0102	2.0		probable carbonic anhydrase	Adaptation, Protection; Central intermediary metabolism
PA0126	2.2		hypothetical protein	Hypothetical, unclassified, unknown
PA0140	3.7	AhpF	alkyl hydroperoxide reductase subunit F	Adaptation, Protection
PA0141	4.9		conserved hypothetical protein	Hypothetical, unclassified, unknown
PA0421	3.7		hypothetical protein	Putative enzymes
PA0578	3.9		conserved hypothetical protein	Hypothetical, unclassified, unknown
PA0840	11.7		probable oxidoreductase	Putative enzymes
PA0848	2.0	AhpB	alkyl hydroperoxide reductase , AhpB	Adaptation, Protection; Putative enzymes
PA0849	5.4	TrxB2	thioredoxin reductase 2	Nucleotide biosynthesis and metabolism
PA0852	11.7	CbpD	chitin-binding protein CbpD precursor	Secreted Factors (toxins, enzymes, alginate)
PA0865	2.2	Hpd	4-hydroxyphenylpyruvate dioxygenase	Amino acid biosynthesis and metabolism
PA0872	4.6	PhhA	phenylalanine-4-hydroxylase	Amino acid biosynthesis and metabolism
PA0936	2.3	LpxO2	lipopolysaccharide biosynthetic protein LpxO2	Cell wall / LPS / capsule; Putative enzymes
PA0997	8.9	PqsB	PqsB	Biosynthesis of cofactors, prosthetic groups and carriers
PA0998	5.8	PqsC	PqsC	Biosynthesis of cofactors, prosthetic groups and carriers
PA0999	2.7	PqsD	3-oxoacyl-[acyl-carrier-protein] synthase III	Biosynthesis of cofactors, prosthetic groups and carriers
PA1023	2.1		probable short-chain dehydrogenase	Putative enzymes
PA1043	4.8		hypothetical protein	Hypothetical, unclassified, unknown
PA1094	2.8	FliD	flagellar capping protein FliD	Cell wall / LPS / capsule; Motility & Attachment
PA1155	4.6	NrdB	NrdB, tyrosyl radical-harboring component of class Ia ribonucleotide reductase	Nucleotide biosynthesis and metabolism
PA1162	2.6	DapE	succinyl-diaminopimelate desuccinylase	Amino acid biosynthesis and metabolism
PA1324	5.6		hypothetical protein	Hypothetical, unclassified, unknown
PA1430	2.3	LasR	transcriptional regulator LasR	Adaptation, Protection; Transcriptional regulators
PA1544	2.1	Anr	transcriptional regulator Anr	Transcriptional regulators
PA1617	3.5		probable AMP-binding enzyme	Putative enzymes
PA1746	2.7		hypothetical protein	Hypothetical, unclassified, unknown
PA1802	2.7	ClpX	ClpX	Cell wall / LPS / capsule; Chaperones & heat shock proteins
PA1816	2.0	DnaQ	DNA polymerase III, epsilon chain	Nucleotide biosynthesis and metabolism
PA1901	6.8	PhzC2	phenazine biosynthesis protein PhzC	Secreted Factors (toxins, enzymes, alginate)
PA1902	10.2	PhzD2	phenazine biosynthesis protein PhzD	Secreted Factors (toxins, enzymes, alginate)
PA1904	4.0	PhzF2	probable phenazine biosynthesis protein	Secreted Factors (toxins, enzymes, alginate)
PA1905	8.5	PhzG2	probable pyridoxamine 5'-phosphate oxidase	Secreted Factors (toxins, enzymes, alginate)
PA1999	2.7	DchA	dehydrocarnitine CoA transferase, DchA	Amino acid biosynthesis and metabolism
PA2000	2.7	DchB	dehydrocarnitine CoA transferase, DchB	Amino acid biosynthesis and metabolism
PA2019	12.2	MexX	Resistance-Nodulation-Cell Division (RND) multidrug efflux membrane fusion protein MexX precursor	Antibiotic resistance and susceptibility; Transport of small molecules
PA2020	6.4	MexZ	MexZ	Transcriptional regulators
PA2069	2.6		probable carbamoyl transferase	Putative enzymes
PA2116	2.4		conserved hypothetical protein	Hypothetical, unclassified, unknown
PA2134	43.0		hypothetical protein	Hypothetical, unclassified, unknown
PA2171	2.4		hypothetical protein	Hypothetical, unclassified, unknown
PA2195	20.6	HcnC	hydrogen cyanide synthase HcnC	Central intermediary metabolism
PA2204	3.2		probable binding protein component of ABC transporter	Transport of small molecules

PA2290	5.0	Gcd	glucose dehydrogenase	Energy metabolism; Carbon compound catabolism
PA2300	2.0	ChiC	chitinase	Carbon compound catabolism
PA2302	2.5	AmbE	AmbE	Putative enzymes; Secreted Factors (toxins, enzymes, alginate)
PA2303	2.9	AmbD	AmbD	Secreted Factors (toxins, enzymes, alginate)
PA2304	5.5	AmbC	AmbC	Secreted Factors (toxins, enzymes, alginate)
PA2366	10.8	HsiC3	HsiC3	Hypothetical, unclassified, unknown
PA2444	2.0	GlyA2	serine hydroxymethyltransferase	Amino acid biosynthesis and metabolism
PA2446	2.3	GcvH2	glycine cleavage system protein H2	Amino acid biosynthesis and metabolism
PA2491	2.6	MexS	MexS	Transcriptional regulators; Putative enzymes
PA2540	2.1		conserved hypothetical protein	Hypothetical, unclassified, unknown
PA2555	2.2		probable AMP-binding enzyme	Putative enzymes
PA2582	2.3		hypothetical protein	Hypothetical, unclassified, unknown
PA2587	8.6	PqsH	probable FAD-dependent monooxygenase	Biosynthesis of cofactors, prosthetic groups and carriers
PA2592	3.3		probable periplasmic spermidine/putrescine-binding protein	Transport of small molecules
PA2622	7.5	CspD	cold-shock protein CspD	Adaptation, Protection; Transcriptional regulators
PA2856	2.3	TesA	lysophospholipase A	Fatty acid and phospholipid metabolism
PA2939	11.6		probable aminopeptidase	Secreted Factors (toxins, enzymes, alginate)
PA2945	2.4		conserved hypothetical protein	Biosynthesis of cofactors, prosthetic groups and carriers
PA2971	2.1		conserved hypothetical protein	Hypothetical, unclassified, unknown
PA3062	2.7	PelC	PelC	Cell wall / LPS / capsule
PA3126	2.0	IbpA	heat-shock protein IbpA	Chaperones & heat shock proteins
PA3141	3.1	WbpM	nucleotide sugar epimerase/dehydratase WbpM	Cell wall / LPS / capsule; Putative enzymes; Membrane proteins
PA3165	3.3	HisC2	histidinol-phosphate aminotransferase	Amino acid biosynthesis and metabolism
PA3328	5.4		probable FAD-dependent monooxygenase	Putative enzymes
PA3329	2.3		hypothetical protein	Hypothetical, unclassified, unknown
PA3330	2.3		probable short chain dehydrogenase	Putative enzymes
PA3331	3.7		cytochrome P450	Adaptation, Protection; Carbon compound catabolism
PA3332	2.0		conserved hypothetical protein	Hypothetical, unclassified, unknown
PA3333	3.2	FabH2	3-oxoacyl-[acyl-carrier-protein] synthase III	Fatty acid and phospholipid metabolism
PA3460	2.1		probable acetyltransferase	Putative enzymes
PA3468	2.1		conserved hypothetical protein	Hypothetical, unclassified, unknown
PA3477	4.2	RhlR	transcriptional regulator RhlR	Transcriptional regulators
PA3539	4.9		conserved hypothetical protein	Hypothetical, unclassified, unknown
PA3622	2.5	RpoS	sigma factor RpoS	Transcriptional regulators
PA3691	2.3		hypothetical protein	Hypothetical, unclassified, unknown
PA3724	3.2	LasB	elastase LasB	Secreted Factors (toxins, enzymes, alginate);Translation, post-translational modification, degradation
PA3792	2.0	LeuA	2-isopropylmalate synthase	Amino acid biosynthesis and metabolism
PA3805	5.8	PilF	type 4 fimbrial biogenesis protein PilF	Motility & Attachment; Protein secretion/export apparatus
PA3806	2.7		conserved hypothetical protein	Hypothetical, unclassified, unknown
PA3819	2.5		conserved hypothetical protein	Membrane proteins
PA3846	7.5		hypothetical protein	Hypothetical, unclassified, unknown
PA3887	3.9	NhaP	Na+/H+ antiporter NhaP	Membrane proteins; Transport of small molecules
PA3925	2.4		probable acyl-CoA thiolase	Putative enzymes
PA3945	4.8		conserved hypothetical protein	Hypothetical, unclassified, unknown
PA3996	4.1	Lis	lipoate synthase	Biosynthesis of cofactors, prosthetic groups and carriers
PA4129	3.2		hypothetical protein	Adaptation, Protection
PA4190	3.6	PqsL	probable FAD-dependent monooxygenase	Putative enzymes
PA4209	4.0	PhzM	probable phenazine-specific methyltransferase	Putative enzymes
PA4211	2.4	PhzB1	probable phenazine biosynthesis protein	Secreted Factors (toxins, enzymes, alginate)
PA4217	3.2	PhzS	flavin-containing monooxygenase	Putative enzymes
PA4241	2.2	RpsM	30S ribosomal protein S13	Translation, post-translational modification, degradation
PA4246	2.2	RpsE	30S ribosomal protein S5	Translation, post-translational modification, degradation
PA4356	2.5	XenB	xenobiotic reductase	Adaptation, Protection; Antibiotic resistance and susceptibility

PA4515	2.2	PiuC	conserved hypothetical protein	Hypothetical, unclassified, unknown
PA4632	2.3		hypothetical protein	Hypothetical, unclassified, unknown
PA4762	2.1	GrpE	heat shock protein GrpE	Chaperones & heat shock proteins; DNA replication, recombination, modification and repair
PA4778	10.5	CueR	CueR	Transcriptional regulators
PA4931	2.1	DnaB	replicative DNA helicase	DNA replication, recombination, modification and repair
PA4963	2.2		hypothetical protein	Hypothetical, unclassified, unknown
PA5220	5.9		hypothetical protein	Hypothetical, unclassified, unknown
PA5279	2.0		conserved hypothetical protein	Hypothetical, unclassified, unknown
PA5285	3.3	SutA	SutA	Hypothetical, unclassified, unknown; Transcriptional regulators
PA5304	2.3	DadA	D-amino acid dehydrogenase, small subunit	Amino acid biosynthesis and metabolism; Energy metabolism
PA5340	2.2		hypothetical protein	Hypothetical, unclassified, unknown
PA5400	5.6		probable electron transfer flavoprotein alpha subunit	Energy metabolism
PA5471	16.2	ArmZ	ArmZ	Antibiotic resistance and susceptibility
PA5475	6.3		hypothetical protein	Hypothetical, unclassified, unknown
PA5481	2.5		hypothetical protein	Hypothetical, unclassified, unknown
PA5528	2.3		hypothetical protein	Membrane proteins
PA5546	2.4		conserved hypothetical protein	Putative enzymes

Supplementary Table 2 List of less differentially abundant proteins in whole cell biofilm in *P. aeruginosa* exposed to tobramycin

Locus Tag	Fold Change	Name	Product Description	PseudoCAP Class category
PA0004	-2.2	GyrB	DNA gyrase subunit B	DNA replication, recombination, modification and repair
PA0041	-2.2		probable hemagglutinin	Secreted Factors (toxins, enzymes, alginate)
PA0155	-2.4	PcaR	transcriptional regulator PcaR	Carbon compound catabolism; Transcriptional regulators
PA0413	-3.5	ChpA	component of chemotactic signal transduction system	Chemotaxis; Motility & Attachment; Two-component regulatory systems
PA0519	-6.0	NirS	nitrite reductase precursor	Energy metabolism
PA0743	-2.4		probable 3-hydroxyisobutyrate dehydrogenase	Carbon compound catabolism
PA0782	-2.2	PutA	proline dehydrogenase PutA	Amino acid biosynthesis and metabolism
PA1135	-2.1	HchA	conserved hypothetical protein	Hypothetical, unclassified, unknown
PA1174	-4.1	NapA	periplasmic nitrate reductase protein NapA	Energy metabolism
PA1327	-3.1		probable protease	Putative enzymes
PA1458	-3.0		probable two-component sensor	Chemotaxis; Two-component regulatory systems
PA1562	-3.8	AcnA	aconitate hydratase 1	Energy metabolism
PA1585	-2.7	SucA	2-oxoglutarate dehydrogenase (E1 subunit)	Amino acid biosynthesis and metabolism; Energy metabolism
PA1737	-2.5		probable 3-hydroxyacyl-CoA dehydrogenase	Putative enzymes
PA1863	-3.7	ModA	molybdate-binding periplasmic protein precursor ModA	Transport of small molecules
PA2147	-5.3	KatE	catalase HPII	Adaptation, Protection
PA2158	-3.0		probable alcohol dehydrogenase (Zn-dependent)	Putative enzymes
PA2160	-3.0		probable glycosyl hydrolase	Putative enzymes
PA2164	-2.0		probable glycosyl hydrolase	Putative enzymes
PA2323	-2.1	GapN	GapN	Carbon compound catabolism
PA2642	-2.4	NuoG	NADH dehydrogenase I chain G	Energy metabolism
PA2699	-2.1		hypothetical protein	Hypothetical, unclassified, unknown
PA2951	-2.2	EtfA	electron transfer flavoprotein alpha-subunit	Energy metabolism
PA2976	-2.2	Rne	ribonuclease E	Transcription, RNA processing and degradation
PA3083	-2.9	PepN	aminopeptidase N	Translation, post-translational modification, degradation
PA3115	-2.7	FimV	Motility protein FimV	Membrane proteins; Motility & Attachment
PA3195	-2.2	GapA	glyceraldehyde 3-phosphate dehydrogenase	Carbon compound catabolism; Energy metabolism
PA3415	-3.0		probable dihydrolipoamide acetyltransferase	Energy metabolism
PA3416	-3.5		probable pyruvate dehydrogenase E1 component, beta chain	Energy metabolism
PA3417	-3.7		probable pyruvate dehydrogenase E1 component, alpha subunit	Energy metabolism
PA3529	-2.8		alkylhydroperoxide reductase C	Adaptation, Protection; Putative enzymes
PA3763	-3.5	PurL	phosphoribosylformylglycinamidine synthase	Nucleotide biosynthesis and metabolism
PA3790	-2.5	OprC	Putative copper transport outer membrane porin OprC precursor	Transport of small molecules
PA3987	-2.2	LeuS	leucyl-tRNA synthetase	Amino acid biosynthesis and metabolism; Translation, post-translational modification, degradation
PA4221	-5.0	FptA	Fe(III)-pyochelin outer membrane receptor precursor	Transport of small molecules
PA4222	-4.6		probable ATP-binding component of ABC transporter	Transport of small molecules
PA4223	-4.9	PchH	probable ATP-binding component of ABC transporter	Membrane proteins; Transport of small molecules
PA4403	-3.0	SecA	secretion protein SecA	Protein secretion/export apparatus
PA4500	-2.4	DppA3	probable binding protein component of ABC transporter	Transport of small molecules

PA4501	-3.4	OpdD	Glycine-glutamate dipeptide porin OpdP	Membrane proteins; Transport of small molecules
PA4502	-3.4	DppA4	probable binding protein component of ABC transporter	Transport of small molecules
PA4560	-3.5	IleS	isoleucyl-tRNA synthetase	Amino acid biosynthesis and metabolism; Translation, post-translational modification, degradation
PA4656	-2.2		conserved hypothetical protein	Hypothetical, unclassified, unknown
PA4675	-2.2	ChtA	ChtA	Transport of small molecules
PA4733	-3.2	AcsB	acetyl-coenzyme A synthetase	Carbon compound catabolism; Central intermediary metabolism
PA4735	-2.9		hypothetical protein	Hypothetical, unclassified, unknown
PA4740	-2.4	Pnp	polyribonucleotide nucleotidyltransferase	Transcription, RNA processing and degradation
PA4756	-2.0	CarB	carbamoylphosphate synthetase large subunit	Amino acid biosynthesis and metabolism; Nucleotide biosynthesis and metabolism
PA4910	-2.9		branched chain amino acid ABC transporter ATP binding protein	Transport of small molecules
PA4915	-2.4		probable chemotaxis transducer	Adaptation, Protection; Chemotaxis
PA4937	-3.5	Rnr	exoribonuclease RNase R	Transcription, RNA processing and degradation
PA5015	-2.7	AceE	pyruvate dehydrogenase	Amino acid biosynthesis and metabolism; Energy metabolism
PA5168	-3.3	DctQ	DctQ	Membrane proteins; Transport of small molecules
PA5373	-2.3	BetB	betaine aldehyde dehydrogenase	Adaptation, Protection; Amino acid biosynthesis and metabolism
PA5506	-2.7		hypothetical protein	Hypothetical, unclassified, unknown
PA5522	-2.4	PauA6	Glutamylpolyamine synthetase	Carbon compound catabolism; Putative enzymes

Supplementary Table 3 HAQs production in biofilm cultures exposed or not to tobramycin of Δ*pqsA* and its isogenic parent strain H103

	**Concentration§ (µg.l⁻¹ of whole biofilm suspension)**		
**Strain**	**HHQ**	**PQS**	**HQNO**
**H103**	82.196 ± 8.572	255.323 ± 22.474	184.568 ± 9.710
**H103-TOB**	121.156 ± 2.098★	947.844 ± 17.395★★	313.187 ± 4.129★★
**Δ*pqsA***	0.058 ± 0.005★★###	0.794 ± 0.102★★###	0.148 ± 0.027★★★###
**Δ*pqsA* -TOB**	0.059 ± 0.017★★NS	1.991 ± 0.356★★★‡	0.282 ± 0.076★★★NS

§ Concentrations of each HAQ in the whole biofilm suspension of the indicated strains grown with or without tobramycin as indicated, were determined by LC-MS/MS and normalized by culture density as described in the Methods section.
Significant differences were determined by a two-tailed *t*-test.
★ indicate the following p values when comparing the H103 wild type strain with tobramycin, ΔpqsA mutant without tobramycin, and ΔpqsA mutant with tobramycin, to the wild type strain: ★$p < 0.05$; ★★$p < 0.01$; ★★★ $p < 0.001$
‡ indicate the following p values when comparing the Δ*pqsA* mutant with and without tobramycin supplementation: ‡$p < 0.05$
# indicate the following p values when comparing the Δ*pqsA* mutant with tobramycin to the H103 wild type strain with tobramycin : ### $p < 0.001$
NS (Not Significative) $p \geq 0.05$

Supplementary Table 4 Bacterial strains and plasmids used in this study

Strain or plasmid	Genotype or relevant characteristics	Reference or source
Pseudomonas aeruginosa PAO1 strains		
H103	Wild-type strain; prototroph derivative of PAO1	Hancock and Carey, 1979
Δ*pqsA*::Gm*lox*	PAO1 H103 Δ*pqsA*:: Gm*lox*; Gm^R	This study
Δ*pqsA*	PAO1 H103 Δ*pqsA::lox*	This study
Δ*prrF*::Gm*lox*	PAO1 H103 Δ*prrF*:: Gm*lox*; Gm^R	This study
Δ*prrF*	PAO1 H103 Δ*prrF::lox*	This study
Escherichia coli strains		
TOP10	Competent cells used as cloning host	Invitrogen
S17.1	*recA pro* (RP4-2Tet::Mu Kan::Tn7), donor and helper strain for conjugation	Simon et al., 1983
Plasmids		
pEX100Tlink	pUC19-based gene replacement vector with MCS[a], sacB; Ap^R	Quénée *et al.,* 2005
pUCGM*lox*	pUC18-based vector containing the *lox* flamked *aacC1* gene; Ap^R, Gm^R	Quénée *et al.,* 2005
pCM157	*cre* expression vector; Tc^R	Marx and Lidstrom, 2002
pEX::Δ*pqsA*	pEX100Tlink containing 5' and 3' flanking sequence of *pqsA* gene; Ap^R	This study
pEX::Δ*pqsA*::Gm*lox*	pEX100Tlink containing 5' and 3' flanking sequence of *pqsA*:: Gm*lox*; Ap^R, Gm^R	This study
pEX::Δ*prrF*	pEX100Tlink containing 5' and 3' flanking sequence of *prrF1,F2* gene; Ap^R	This study
pEX::Δ*prrF*::Gm*lox*	pEX100Tlink containing 5' and 3' flanking sequence of *prrF*:: Gm*lox*; Ap^R, Gm^R	This study

[a], multiple cloning site

[R], antibiotic resistance

E. coli strains were grown in the presence of ampicillin (Ap; 100 µg.ml^{-1}), gentamicin (Gm; 15 µg.ml^{-1}) or tetracycline (Tc; 10 µg.ml^{-1}).

P. aeruginosa strains were grown in LB liquid medium in the presence of carbenicillin (Cb; 300 µg.ml^{-1}), Gm (30 µg.ml^{-1}), or Tc (125 µg.ml^{-1}) or on LB agar medium containing Cb (600 µg.ml^{-1}), Gm (100 µg.ml^{-1}), or Tc (250 µg.ml^{-1})

Supplementary References

Hancock, R. E. W. & Carey, A. M. Outer membrane of *Pseudomonas aeruginosa*: Heat- and 2-mercaptoethanol-modifiable proteins. *J. Bacteriol.* **140**, 902–910 (1979).

Marx, C. J. & Lidstrom, M. E. Broad-host-range *cre-lox* system for antibiotic marker recycling in Gram-negative bacteria. *BioTechniques* **33**, 1062–1067 (2002).

Quénée, L., Lamotte, D. & Polack, B. Combined *sacB*-based negative selection and *cre-lox* antibiotic marker recycling for efficient gene deletion in *Pseudomonas aeruginosa. BioTechniques* **38**, 63–67 (2005).

Simon, R., Priefer, U. & Pühler, A. A broad host range mobilization system for in vivo genetic engineering: Transposon mutagenesis in Gram negative bacteria. *Bio/Technology* **1**, 784–791 (1983).

Supplementary Table 5 List of primers used in this study

Gene name	Sequence (5' > 3')[a]	Length
Construction of the *pqsA* mutant		
pqsA-SacI-F	taataa-**GAGCTC**-CTGCACGGCTATTTCCGTAT	32
pqsA-XbaI-R	taataa-**TCTAGA**-CAGAACGTTCCCTCTTCAGC	32
pqsA-XbaI-F	taataa-**TCTAGA**-CATGTTGATTCAGGCTGTGG	32
pqsA-HindIII-R	taataa-**AAGCTT**-CGCTGCCAGTTTGACCTTAT	32
Construction of the *prrF* mutant		
prrF-EcoRI-F	taataa-**GAATTC**-TGGACGTTCGTACCTCCTCT	32
prrF-XbaI-R	taataa-**TCTAGA**-TTTCGCCAAACTTTTCTGCT	32
prrF-XbaI-F	taataa-**TCTAGA**-TTCCTTCAACGTGCCTTCTT	32
prrF-HindIII-R	taataa-**AAGCTT**-TGTTCGCTTTCTGTCTGCTG	32
Quantification of mRNA levels by RT-qPCR		
feoB-F	GATCTTCATCGACGGCATCCAGTG	24
feoB-R	CAGCGAGAGGAACAGGTACATCA	23
hasI-F	TGGATGCCGATGCGCTTG	18
hasI-R	CAGCGGGAATCCTCGAGT	18
phuR-F	AGCCACTCCTGGTTGACCTC	20
phuR-R	CGTAGTTCCAGCCCAGCTTG	20
prrF-F	GCGAGGCCAGCAGGTAAG	18
prrF-R	CGTGATTAGCCTGATGAGGAGAT	23
pvdH-F	CAGCACCATCCTGTCGTTCCA	21
pvdH-R	GCAGGTTCGCCTTGACCC	18
pvdS-F	GGAACAACTGTCTACCCGCA	20
pvdS-R	GTAGCTGAGCTGTGCCTTGA	20
rpoD-F	AGGCCGTGAGCAGGGATAC	19
rpoD-R	TCCCCATGTCGTTGATCATG	20
16S-F	AACCTGGGAACTGCATCCAA	20
16S-R	CTTCGCCACTGGTGTTCCTT	20

[a]All the primers used in this study were synthesized by Eurogentec and are based on *P. aeruginosa* PAO1 genome sequence (http://www.pseudomonas.com).
Bold nucleotides indicate restriction endonuclease sites inserted within primer sequences.
